# Shadow glass transition as a thermodynamic signature of β relaxation in hyper-quenched metallic glasses

**DOI:** 10.1093/nsr/nwaa100

**Published:** 2020-05-13

**Authors:** Qun Yang, Si-Xu Peng, Zheng Wang, Hai-Bin Yu

**Affiliations:** Wuhan National High Magnetic Field Center and School of Physics, Huazhong University of Science and Technology, Wuhan 430074, China; Wuhan National High Magnetic Field Center and School of Physics, Huazhong University of Science and Technology, Wuhan 430074, China; Key Laboratory for Liquid-Solid Structural Evolution and Processing of Materials (Ministry of Education), Shandong University, Jinan 250061, China; Wuhan National High Magnetic Field Center and School of Physics, Huazhong University of Science and Technology, Wuhan 430074, China

**Keywords:** metallic glass, secondary relaxation, shadow glass transition, fast scanning calorimetry

## Abstract

One puzzling phenomenon in glass physics is the so-called ‘shadow glass transition’ which is an anomalous heat-absorbing process below the real glass transition and influences glass properties. However, it has yet to be entirely characterized, let alone fundamentally understood. Conventional calorimetry detects it in limited heating rates. Here, with the chip-based fast scanning calorimetry, we study the dynamics of the shadow glass transition over four orders of magnitude in heating rates for 24 different hyper-quenched metallic glasses. We present evidence that the shadow glass transition correlates with the secondary (β) relaxation: (i) The shadow glass transition and the β relaxation follow the same temperature–time dependence, and both merge with the primary relaxation at high temperature. (ii) The shadow glass transition is more obvious in glasses with pronounced β relaxation, and *vice versa*; their magnitudes are proportional to each other. Our findings suggest that the shadow glass transition signals the thermodynamics of β relaxation in hyper-quenched metallic glasses.

## Teaser

A puzzling shadow glass transition, prior to the real glass transition, is disclosed to correlate with the secondary relaxation in metallic glasses.

## INTRODUCTION

Glasses are disordered materials that lack the long-range order of crystals but behave mechanically like solids, and they are usually prepared by fast cooling from liquids to avoid crystallization [1–9]. Compared to their crystalline counterparts, glass materials are at non-equilibrium states [4,10–13]. When heated from low temperature (e.g. by differential scanning calorimetry, DSC), they exhibit complex relaxation processes before the glass transition temperature (*T_g_*) [14,15]. Specifically, by heating of a rapid quenched glass, it exhibits a pronounced exothermic (heat-releasing) process as a result of aging or structural relaxations, which is usually denoted as the enthalpy relaxation [6,15–18]. On the other hand, if the glass is properly annealed, an additional endothermic (heat-absorbing) peak might show up during the DSC measurement [15,19–24]. As this process resembles the real glass transition in several aspects, it is called ‘shadow glass transition’ or ‘sub-*T_g_* prepeak’ [15,25]. Several previous works have demonstrated that both enthalpy relaxation and shadow glass transition have pronounced effects on the structure-property relations in glasses materials relevant to their glass forming ability, mechanical and magnetic properties [6,26–28], anomalous liquid-properties (e.g. liquid–liquid transition or fragile–strong transition) [15,17,20,29,30], and the correct assignment of *T_g_* in amorphous water and phase-change materials [16,23,25].

While the exothermic enthalpy relaxation might be understood as the continuous transformation of a high enthalpy state to a lower one during slow heating, the endothermic shadow glass transition is intriguing: it seems to indicate that during annealing, some parts of the glass reach lower energy states relative to the rest of the system and then return to the higher energy states during DSC up-scan [3,31]. Some researchers proposed that the shadow glass transition might also imply structural heterogeneity of the glass [15,21,31,32]. The basic question remains unclear as to what kind of atomic motions are responsible for the heating-absorbing shadow glass transition.

Aside from these non-equilibrium relaxation phenomena, glasses and supercooled liquids also have a range of inherent dynamic processes which can be found in both the thermodynamic equilibrium states (the supercooled liquids) and the out-of-equilibrium glass states [33–42]. Among them, the most prominent is the so-called primary (α) relaxation. Its evolution from equilibrium to out-of-equilibrium during cooling of the liquid is associated with the thermodynamic signature of glass transition, as can be measured from the jump of specific heat, Δ*C_p_* [15,39,43]. Processes occurring in addition to the α relaxation at shorter timescales or lower temperature are referred to as secondary (β) relaxations [33,36,42,44,45]. Usually the β relaxations are probed by dielectric or mechanical spectroscopy [36,42,46–55], but could not be readily detected by ordinary DSC procedures. Nevertheless, Fujimoi and Oguni reported thermodynamic signatures of β relaxations by adiabatic calorimetry [56] and Busch *et al.* by the temperature-modulated DSC [19,22]. Recently, Ngai and coworkers, in a series of papers, also proposed other signatures for β relaxations [57,58].

In light of these studies, it is of interest to know whether the shadow glass transition is connected to β relaxations, just as the (real) glass transition is to α relaxations. This question is of crucial importance for both revealing the origin of the shadow glass transition and β relaxation in glassy materials, as well as improving our understanding about the nature of the glass. We note that there are some previous studies that attempted to establish connections between the β relaxation and the (heat-releasing) enthalpy relaxation [6,18,32,59–61]. For instance, the enthalpy relaxation has been considered as a proxy of β relaxation [18], and the activation energy of enthalpy relaxation and β relaxation reported to be nearly equal in some glasses [60]. Logically, on the other hand, by comparing the real glass transition and the α relaxation, one may envisage that if the β relaxation has thermodynamic consequence, it might show an endothermic (heat-absorbing) feature. The shadow glass transition might be such a candidate [62]. Some authors have inferred that the shadow glass transition might be related to the β relaxation based on the activation energy [19,22,25,63]. As these studies depend on the dedicated annealing treatments and as the accessible observation time window is narrow as it is limited by the heating rates of DSC (typically 0.1–1 K/s) [15,18,24,61], it is still difficult to make direct comparisons between the shadow glass transition and the β relaxation. Consequently, whether the shadow glass transition and β relaxation are connected is still not elucidated.

In this work, we use a chip-based fast scanning calorimetry (FSC) [64–74] to investigate the dynamics of the shadow glass transition in a wide range of heating rates (3–20 000 K/s) in two dozen different metallic glasses (MGs). We show that the FSC can clearly capture the shadow glass transition without the need for annealing at high heating rates for rapidly quenched MGs. We illustrate that the dynamics of the shadow glass transition quantitatively match the β relaxation as independently measured by mechanical relaxations. Interestingly, we find that the shadow glass transition is more obvious in glasses with pronounced β relaxation, while it is hard to observe in glasses with weak β relaxation. Our results provide clear evidence on the correlation between the shadow glass transition and the β relaxation. These findings suggest that the shadow glass transition signals the thermodynamic freezing of β relaxation, analogous to the glass transition and the freezing of α relaxation.

## RESULTS

**Figure 1.**
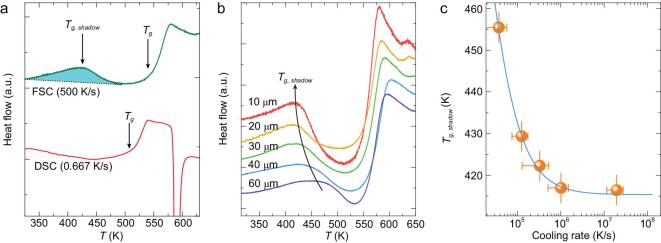
Shadow glass transition of La_50_Ni_15_Co_2_Al_33_ MG. (a) Comparison of heat flow curve at low heating rate (conventional DSC) and high heating rate (FSC). (b) FSC heat flow curves of the La_50_Ni_15_Co_2_Al_33_ alloy for ribbon thickness range from 10 um to 60 um, measured with a heating rate of 500 K/s. (c) The effect of cooling rates on *T_g__, shadow_*.

Figure 1a compares two typical heat flow curves of a La_50_Ni_15_Co_2_Al_33_ MG measured by a conventional DSC (at a heating rate *Q* = 0.333 K/s or 20 K/min) and an FSC (*Q* = 500 K/s), respectively. The conventional DSC curve only exhibits an exothermic process (the enthalpy relaxation) before *T_g_*. In contrast, the FSC curve exhibits a clear endothermic peak, which is the shadow glass transition, in addition to the enthalpy relaxation and the glass transition. We define *T_g__, shadow_* as the temperature corresponding to the maximum point of this endothermic peak. We consider that the shadow glass transition is not a true glass transition, and it does not have a step-like heat-capacity jump. Instead, the shadow glass transition might be better viewed as an activation processes, and thus the peak temperature might be more suitable for analysis than the onset temperature, as is the case for many other activation processes. We note that previous studies of the shadow glass transition have resorted to dedicated thermal annealing procedures [19,20,22,23]. Thus, the FSC enable us to directly investigate the shadow glass transition without the need of annealing.

Figure 1b presents the heat flow curves for five different glassy ribbon samples with thickness ranging from 10 to 60 μm that are produced by different roller speeds during spinning quenching. Consequently, they have different cooling rates, and the thinner the sample, the higher the cooling rate. Figure 1b indicates that the cooling rate influences the shadow glass transition, as *T_g__, shadow_* decreases with cooling rates. Quantitatively, we estimate the cooling rates of the samples according to the energy matching method of Liu *et al.* [18]. Figure 1c shows the *T_g__, shadow_* as a function of the estimated cooling rate. It reveals that for samples prepared with faster cooling rates, the shadow glass transition can shift to a lower temperature. Interestingly, when the cooling rate is faster than ∼10^6^ K/s, *T_g__, shadow_* gradually approaches a value of constant, as further increasing of the cooling rates does not lead to lowering *T_g__, shadow_* within the experimental sensitivity. Thus, the *T_g__, shadow_* could be used as a materials property only if the samples are prepared by a cooling rate higher than 10^6^ K/s, that is the hyper-quenched glasses. In the following experiments, all the samples are prepared by the highest cooling rates (i.e. with thickness ∼10 μm, or cooling rates larger than 10^6^ K/s).

**Figure 2.**
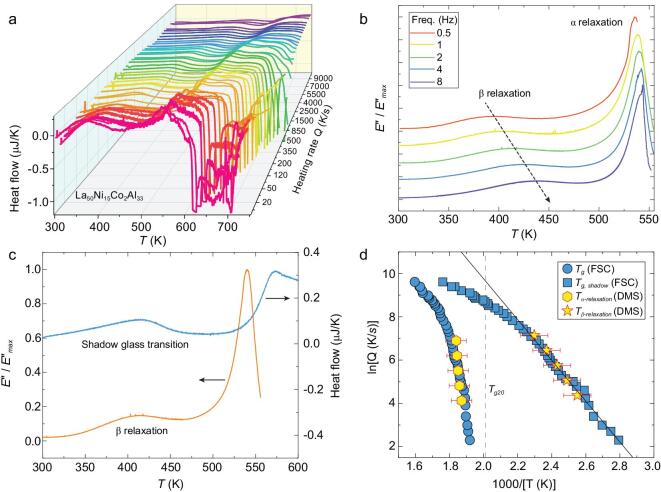
Shadow glass transition and β relaxation in La_50_Ni_15_Co_2_Al_33_ MG. (a) Shadow glass transition of glass ribbon measured at different heating rates. (b) Temperature dependent normalized *E’’*/*E’’_max_* at different testing frequencies. (c) Temperature dependence of the DMS normalized loss modulus (2 Hz) versus FSC heat flow (300 K/s). (d) Relaxation map showing the β relaxation, α relaxation, shadow glass transition and real glass transition as a function of inverse temperature. The *T_g20_*(i.e. glass transition temperature at a heating rate of 20 K/min, as usually set in experiments) is marked by the vertical gray dashed line. The black solid line is the Arrhenius equation fitting to the β relaxation.

Figure 2a presents the typical FSC curves showing heat flow versus temperature at a range of heating rates from 10 to 10 000 K/s for the La_50_Ni_15_Co_2_Al_33_ MG. The dynamic behavior of the shadow glass transition is similar to the real glass transition process, moving to higher temperatures at higher heating rates, which demonstrates that the shadow glass transition is of kinetic nature. Meanwhile, dynamic mechanical spectra (DMS) were carried out at different testing frequencies to investigate its inherent relaxation dynamics. Figure 2b shows the temperature dependence of the normalized loss modulus *E’’*/*E’’_max_* at different testing frequencies for La_50_Ni_15_Co_2_Al_33_ MG. The MG shows pronounced β relaxation peak, in addition to the α relaxation.

Figure 2c shows the FSC heat flow curve (300 K/s) and the normalized loss modulus *E’’*/*E’’_max_* (2 Hz). These two curves are selected due to the glass transition probed by FSC at this heating rate and the α relaxation of DMS at this frequency have nearly the same temperature (∼528 K here). From DMS, one can see a distinct β relaxation peak which locates about 410 K (i.e. the β relaxation peak temperature, *T*_β_ = 410 K). At the same time, we find the FSC curve also exhibits a pronounced endothermic peak in the same temperature range due to the shadow glass transition. In Fig. 2d, we summarized the β and α relaxations from DMS, the shadow glass transition and the (real) glass transition from FSC in a relaxation map for La_50_Ni_15_Co_2_Al_33_ MG. We note that the timescale is represented by two different quantities in the two experiments, namely, the testing frequency (Hz or s^−1^) in DMS and the heating rate (K/s) in FSC. To translate the frequency in DMS to heating rates in FSC, we assume there is a linear relation between them and we vertically shift the DMS data in Fig. 2d to make the α relaxation maximally overlap with the *T_g_* data (at different heating rates) by FSC. The shift-factors are given in the online supplementary data. Importantly, we find that, as shown in Fig. 2d, once the α relaxation is overlapped with *T_g_* (by FSC) by this manipulation, the β relaxation coincides nicely with shadow glass transition as well.

Meanwhile, both the β relaxation peak and shadow glass transition peak can be fitted by an Arrhenius equation at low temperatures. However, with the further increase of heating rate the *T_g__, shadow_* does not follow an Arrhenius behavior for temperatures above *T_g_*, but it follows a super-Arrhenius behavior at a higher temperature and eventually merges into α relaxation (real glass transition) at heating rates above 10 000 K/s. These behaviors are indeed similar to the β relaxation in general. Due to the limited frequency range of our DMS, the β relaxation at higher frequency (or higher temperature) could not be measured in MGs. Nevertheless, several experiments based on dielectric spectroscopy have shown that the β relaxation in molecular glasses merges with the α relaxation in a super-Arrhenius manner. Thus the shadow glass transition behaves like the β relaxation in dynamics.

**Figure 3.**
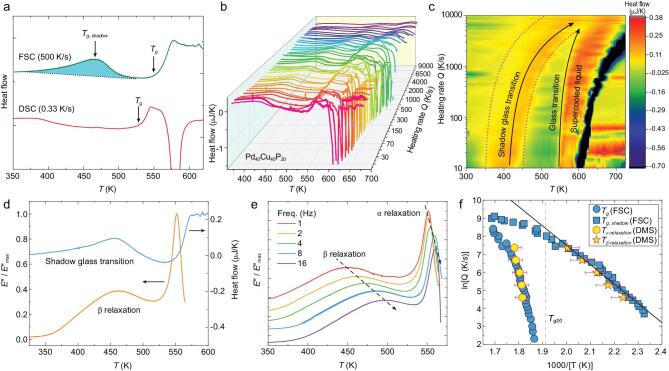
Shadow glass transition and β relaxation in Pd_40_Cu_40_P_20_ MG. (a) Comparison of heat flow curves between conventional DSC and FSC. (b, c) Effect of heating rates on shadow glass transitions. (d) The DMS loss modulus (2 Hz) versus FSC heat flow (200 K/s). (e) The loss modulus curve evolves with different test frequencies. (f) Relaxation map showing the β relaxation, α relaxation, shadow glass transition and real glass transition as a function of inverse temperature.

Similar experiments were also performed for a Pd_40_Cu_40_P_20_ MG. As shown in Fig. 3a, the FSC curve exhibits a clear shadow glass transition at a temperature below the enthalpy relaxation and the *T_g_*. Figure 3b and c shows the heat flow curves of Pd_40_Cu_40_P_20_ MG measured by FSC over a range of heating rates *Q* from 10 to 10 000 K/s. The DMS loss modulus (2 Hz) and the FSC heat flow (200 K/s) are shown in Fig. 3d. Figure 3e shows the dynamic behavior of α relaxation and β relaxation at different test frequencies. The corresponding relaxation map are reported in Fig. 3f which summarizes *T_g__,__shadow_* from FSC and *T_β_* from DMS at different testing frequencies. Again, one can see that the shadow glass transition and β relaxation agree with each other and they also agree with an Arrhenius equation at low temperatures (or heating rates lower than ∼4 000 K/s). As heating rate *Q* increases, the shadow glass transition progressively shifts to a higher temperature at a faster speed, thus, the shadow glass transition follows a super-Arrhenius behavior at a higher heating rate *Q* ≥ 4000 K/s, until it eventually merges with α relaxation near 10 000 K/s. This observation demonstrates again an intrinsic correlation between the shadow glass transition and β relaxation in metallic glasses.

To further verify the above findings, we investigate another six different MGs with pronounced β relaxations as probed by DMS. These are Au_49_Ag_5.5_Pd_2.3_Cu_26.9_Si_16.3_ (Fig. S2), La_65_Ni_20_Al_15_ (Fig. S3), La_65_Cu_20_Al_15_ (Fig. S4), Ce_65_Ni_18_Cu_2_Al_15_ (Fig. S5), Pd_40_Ni_10_Cu_30_P_20_ (Fig. S6) and Ce_65_Ni_10_Al_25_ (Fig. S7(a)). As detailed in Figs S2–S7, they all exhibit the same behaviors with La_50_Ni_15_Co_2_Al_33_ (Fig. 2) and Pd_40_Cu_40_P_20_ (Fig. 3). Thus, a similar conclusion can be obtained for these MGs, which is that there is an intrinsic correlation between the shadow glass transition and the β relaxation in these hyper-quenched MGs.

**Figure 4.**
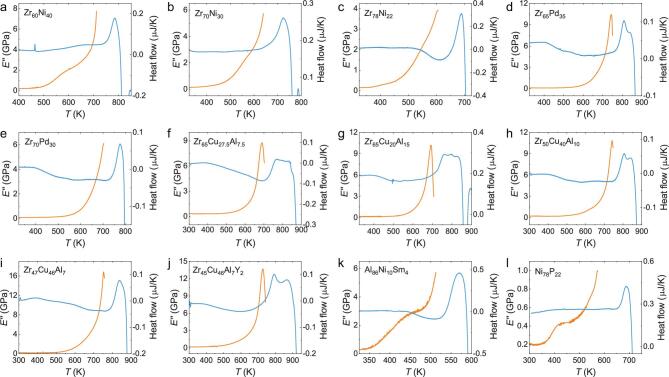
Shadow glass transitions are hardly to be probed in MGs without pronounced β relaxation. (a–I) Temperature dependence of the DMS loss modulus *E’’* (dark yellow, left axis) and FSC heat flow (blue, right axis) measured with a heating rate of 500 K/s for 12 different MGs with compositions indicated.

Previous studies have shown that the behaviors of β relaxation are materials specific and sensitive to chemical compositions [36,42,75]. In some MGs, β relaxations manifest as distinct peaks, while in some other systems, β relaxations appear to be absent and, instead, excess contributions to the tails of α relaxations show up [36,37,42,54,76,77]. These so-called excess wings have been observed in many systems without well-resolved peaks of β relaxations [36,42,77]. Since the above experiments were conducted in MGs with pronounced β relaxations, it is of interest to study the effect of the unobvious β relaxation (e.g. shoulder or excess wings) on shadow glass transition. We therefore investigate the FSC and DMS on Ni_78_P_22_, Al_86_Ni_9_Sm_5_ and 13 different Zr-based MGs (Table 1). What is common to these MGs is that they do not have pronounced β relaxations. They either show excess wings or shoulder-like features as probed by DMS. Figure 4 shows the temperature dependence of the DMS loss modulus (1 Hz) and the FSC heat flow (500 K/s) for these MGs. One can see that none of them exhibits a clear shadow glass transition as probed by FSC. This result suggests that the magnitudes of shadow glass transition and the β relaxation evolve hand in hand with each other, providing more evidence as to correlation between them.

**Table 1.** Cross-correlation between the behavior of the β relaxation and shadow glass transition for 24 different metallic glasses.

	Shadow *T _g_*	
β relaxation	Observed	Not observed
Peak or pronounced hump	Pd_40_Cu_40_P_20_	
	La_50_Ni_15_Co_2_Al_33_	
	La_65_Ni_20_Al_15_	
	Pd_40_Ni_10_Cu_30_P_20_	
	Au_49_Ag_5.5_Pd_2.3_Cu_26.9_Si_16.3_	
	Ce_65_Ni_10_Al_25_	
Shoulder	La_65_Cu_20_Al_15_	Al_86_Ni_10_Sm_4_
	Pd_40_Ni_40_P_20_	Ni_78_P_22_
	Ce_65_Ni_18_Cu_2_Al_15_	Zr_70_Ni_30_
		Zr_60_Ni_40_
Excess wing		Zr_78_Ni_22_
		Zr_50_Cu_40_Al_10_
		Zr_65_Cu_27.5_Al_7.5_
		Zr_65_Cu_20_Al_15_
		Zr_47_Cu_46_A_7_
		Zr_45_Cu_46_Al_7_Y_2_
		Zr_63_Cu_20_Al_15_Y_2_
		Zr_70_Pd_30_
		Zr_65_Pd_35_
		Zr_60_Ni_25_Al_15_
		Zr_46_Cu_39_Al_8_Ag_7_

The results for all the studied MGs are collectively shown in Table 1, where the MGs are classified into different groups by two features: the behavior of the β relaxation in each row and the shadow glass transition in each column. We can see that the shadow glass transition is always found in the hyper-quenched MGs with pronounced β relaxation. On the other hand, the MGs without obvious β relaxation are less likely to show shadow glass transition as probed by FSC.

**Figure 5.**
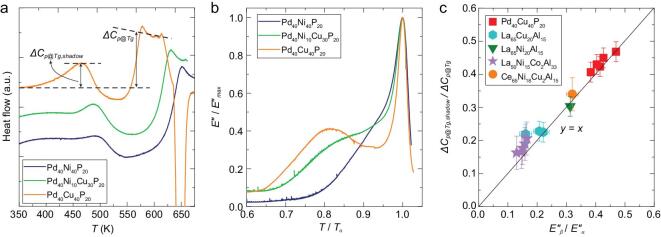
Relative strength of shadow glass transition and β relaxation. (a) Temperature dependence of the FSC heat flow for the Pd_40_Ni_40-_*_x_*Cu*_x_*P_20_ (0, 30 and 40) MGs at the heating rate of 500 K/s. (b) Temperature dependence of *E’’/E’’_max_* for the Pd_40_Ni_40-_*_x_*Cu*_x_*P_20_ (0, 30 and 40) MGs at the testing frequency of 1 Hz. *E’’/E’’_max_* is normalized by the loss modulus *E’’* at *T_α_*. (c) Relationship between *ΔC_p@Tg__,__shadow_/ΔC_p@Tg_* and *E’’_β_*/*E’’_α_*.

To quantitatively correlate the distinct behaviors of β relaxation and the shadow glass transition, the relative heights of β relaxation and shadow glass transition can be determined respectively as *E’’_β_/E’’_α_* and *ΔC_p@Tg__,__shadow_/ΔC_p@Tg_*. Here, *E’’_β_/E’’_α_* is the ratio between peak height of β relaxation and α relaxation. Similarly, *ΔC_p@Tg__,__shadow_/ΔC_p@Tg_* is the ratio between the peak height of shadow glass transition *ΔC_p@Tg__,__shadow_* and the heat capacity jump of real glass transition *ΔC_p@Tg_*. Here, we first use the Pd-based MGs system as a typical example to illustrate the relation between the shadow glass transition and β relaxation. One can see a trend that the *ΔC_p@Tg__,__shadow_/ΔC_p@Tg_* increase with the addition of the Cu into Pd_40_Ni_40_P_20_ MG to replace Ni atom for Pd_40_Ni_40-_*_x_*Cu*_x_*P_20_ (*x* = 0, 30 and 40) MGs system, as shown in Fig. 5a. At the same time, when Cu is added into Pd_40_Ni_40_P_20_ to replace Ni, the peaks of β relaxation also shift gradually to lower-scaled temperatures and become more pronounced as shown in Fig. 5b. In other words, alloying influences in the same way to the relative strength of β relaxation and the shadow glass transition.

Figure 5c presents the quantitative relationship between the β relaxation and the shadow glass transition by plotting *ΔC_p@Tg__,__shadow_/ΔC_p@Tg_* against *E’’_β_/E’’_α_*. It is noteworthy that *ΔC_p@Tg__,__shadow_/ΔC_p@Tg_* is nearly a proportional (i.e. *y* = *x*) function of *E’’_β_*/*E’’_α_* for these MGs. It indicates that the stronger shadow glass transition with higher *ΔC_p@Tg__,__shadow_/ΔC_p@Tg_* corresponds to a more pronounced β relaxation peak and *vice versa*. This corroborates that the strength of shadow glass transition and the behaviors of β relaxation are correlated.

## DISCUSSION

These results inspire the physical mechanism that a β relaxation induced connectivity percolation happens before the glass transition and leads to the sub-*T_g_* endothermic peak. The β relaxation in MGs has been identified to reflect the string-like collective atomic arrangement based on molecular dynamics simulations [51,78,79]. Previous experiments also found that the fraction of liquid-like regions (or ‘flow units’) was above 0.25 after the full activation of β relaxation [62,80,81]. The value between 0.25–0.3 happens to be the threshold volume fraction of connectivity percolation for a 3D continuum system [82–84]. The connectivity percolation means that the expansion of activated liquid-like regions with increasing temperature enables the appearance of at least one connected flow unit chain to penetrate through the sample. Unlike the ‘real’ glass transition, where we believe a rigidity percolation happens and the sample behaves with a macroscopic softness, the ‘shadow’ glass transition is rather confined with no additional macroscopic degree of freedom. Therefore, an endothermic peak which reflects the local to cooperative transition can be observed but with a smaller value compared to a ‘real’ glass transition. However, it is a kinetic process in the real world and the competition between the activation process and structural relaxation will weaken the endothermic process if the heating rate is slow. This explains the reason why the shadow glass transition peak is difficult to detect by using traditional calorimetry equipment. If the sample is heated up fast enough, the connectivity and rigidity percolation may be reached simultaneously and the shadow glass transition will merge into the main glass transition as shown in Figs 2d, and 3c and f.

Besides, the energy status of sample or chemical influence also plays an important role in the activation process. Generally, the low cooling rate and annealing treatment will lower both the system energy and the diversity of structural heterogeneity, which means the connectivity percolation can only be reached at a higher temperature. From our FSC results, lower cooling rate indeed leads to a higher shadow glass transition as predicted from the model. Chemical influence on shadow glass transition is as strong as on β relaxation, where no clear shadow glass transition can be probed even by FSC in systems with weak β relaxation behaviors. The physical mechanism for the phenomenon might also be related to the percolation state. The unobvious β relaxation shoulder or excess wing is believed to result from the indiscernibility between the two relaxations, where deduced *T_β_* is close to 0.9*T_α_* (here, *T*_α_ is the peak temperature of the α relaxation) and therefore β peak hidden in the flank of α peak [85]. Weak β relaxation behavior together with fewer flow unit regions will result in an undistinguished shadow glass transition, which was observed in those Zr-, Ni- and Al-based MGs (Fig. 4).

We have shown that the shadow glass transition and β relaxation follow a same temperature-time dynamic and their magnitudes are proportional with each other. These results are enabled by the combined experiments of dynamical mechanical analysis and, especially, the recently developed fast-scanning calorimetry with heating rates of hundreds/thousands kelvin per second. Our findings establish a correlation between the two seemingly different processes, which provides an example of settling long-standing attempts to relate glass dynamics to thermodynamic responses. Meanwhile, the progress in the understanding of β relaxation could be suggestive of ultimately resolving the mechanisms of shadow glass translation. The emerging physical picture implies that the shadow glass transition is a thermodynamic signature of β relaxation in hyper-quenched glasses, analogous to the glass transition and the freezing of α relaxation. The results presented above thus open new challenges and opportunities for furthering our understanding of glass relaxations.

## METHODS

### Sample preparation

We selected 24 different MGs for experiments based on their different relaxation behaviors. The chemical compositions of them are listed in Table 1. The initial Pd_40_Cu_40_P_20_, Pd_40_Ni_40_P_20_, Pd_40_Ni_10_Cu_30_P_20_ and Ni_78_P_22_ alloy ingots were prepared by induction melting of high purity elements under an argon-purged atmosphere; Pd (99.99 at%), Ni (99.99 at%), Cu (99.99 at%) and red phosphorus powder (98.5 at%). The resulting Pd_40_Cu_40_P_20_, Pd_40_Ni_40_P_20_, Pd_40_Ni_10_Cu_30_P_20_ and Ni_78_P_22_ alloys were treated with B_2_O_3_ flux for 3 h. Ingots of the Au_49_Ag_5.5_Pd_2.3_Cu_26.9_Si_16.3_, La_65_Ni_20_Al_15_, La_65_Cu_65_Al_15_, La_50_Ni_15_Co_2_Al_33_, Ce_65_Ni_10_Al_25_, Ce_65_Ni_18_Cu_2_Al_15_, Al_86_Ni_10_Sm_4_ and other Zr-based alloys were prepared by melting high purity elements (purity ≥ 99.95 at%) under a Ti-gettered argon atmosphere in an arc-melting furnace. The ingots were re-melted five times to ensure compositional homogeneity. Amorphous ribbons, about 20 um thick and 3 mm wide, were prepared by re-melting the alloys using rf induction and injecting the melts onto the surface of a single copper roller with the speed of 50–65 m/s for these different alloy compositions. Amorphous ribbons of different thicknesses are achieved by varying the rotational speed of the rollers at speeds between 20 and 70 m/s for La_50_Ni_15_Co_2_Al_33_ MG. The glassy nature of all the ribbons was verified using X-ray diffraction (XRD, Bruke D2 phaser) with monochromatic Cu Kα radiation (λ = 0.1542 nm) and DSC (Mettler Toledo DSC 3).

### Dynamical mechanical analysis

The dynamical mechanical spectra of these MGs were measured on a TA Q800 dynamical mechanical analyzer. For these amorphous ribbon samples, film tension mode was used in an isochronal mode with a heating rate of 3 K/min, strain amplitude of 6 um and discrete testing frequency of 0.5, 1, 2, 4, 8 and 16 Hz.

### Calorimetry measurements

The present calorimetry was performed using a combination of Flash DSC (Mettler Toledo Flash DSC 2+) and conventional DSC (Mettler Toledo DSC 3). The heat flow curves of MGs at a relatively low heating rate (0.083–1.33 K/s) is obtained by continuous heating on a conventional DSC using a refrigerated cooling system with a N_2_-gas DSC cell purge under a 50 ml/min nitrogen gas flow. The sample masses were 8–15 mg. In order to ensure the reliability of the measurement, each crystallized sample was heated again to obtain a baseline. The conventional DSC was calibrated by using pure In and Zn standard. The heat flow curves of MGs at higher heating rates were obtained by continuous heating on a Flash DSC under 80 ml/min argon gas flow. The twin-type chip sensor based on MEMS technology is made of a sample and a reference. The FSC chip sensors were preconditioned and calibrated following the manufacturer recommendation. The FSC samples were prepared by cutting the melt-spun ribbons into small pieces under a stereomicroscope and then transferred using an electrostatic manipulator hair onto a temperature-corrected MultiSTAR UFS1 sensor or UFH sensor. Samples were placed on the sensitive area of a MEMS chip sensor for a range of heating rates from 3 to 20 000 K/s.

## Supplementary Material

nwaa100_Supplemental_File
