# Supplementary material for: Shadow glass transition as a thermodynamic signature of β relaxation in hyper-quenched metallic glasses
Source: Natl Sci Rev. 2020 May 13;7(12):1896–905. doi: 10.1093/nsr/nwaa100 (PMC8288642; doi:10.1093/nsr/nwaa100)
Supplement: nwaa100_Supplemental_File [file nwaa100_supplemental_file.docx]

**Supplementary Materials for**

**Shadow glass transition as a thermodynamic signature of β relaxation in hyper-quenched metallic glasses**

Qun Yang^1^, Si-Xu Peng^1^, Zheng Wang ^2,^* and Hai-Bin Yu ^1,^*

^1^Wuhan National High Magnetic Field Center and School of Physics, Huazhong University of Science and Technology, WuHan 430074, China

^2^Key Laboratory for Liquid-Solid Structural Evolution and Processing of Materials (Ministry of Education), Shandong University, Jinan 250061, China

*Corresponding authors, Email: [wangzhenglofty@gmail.com](mailto:wangzhenglofty@gmail.com) or [haibinyu@hust.edu.cn](mailto:haibinyu@hust.edu.cn)

The Supplementary Materials provides details on the following three aspects of the work:

1. **Determination of cooling rates (*Q_C_*) of different thickness ribbons.**
2. **Additional data for the relation between shadow glass transition and β relaxation in glasses.**
3. **The shift factors (*n_s_*) for different composition metallic glasses.**

**1. Determination of cooling rates (*Q_C_*) of different thickness ribbons****.**

According to the energy matching method [^1^](#_ENREF_1)^,^ [^2^](#_ENREF_2), *T_f_* value of different thickness ribbons can be evaluated from their DSC heat flow curve under heating rate of 40 K/min. In order to obtain the corresponding cooling rate of *T_f_* value for different thickness ribbons, *T_f_* with known cooling rates is also calculated using energy matching method. Finally, the Arrhenius plot of scaled fictive temperature *T_f_* /*T^s^_f_* and cooling rate *Q*/*Q_s_* as showed in Supplementary Fig. S1, here, *T^s^_f_* and *Q_s_* is fictive temperature and cooling rate for a standard sample (*Q_s_* = 20 K/min), respectively. Therefore, the corresponding cooling rate *Q_C_* can be obtained for different thickness ribbons [^3^](#_ENREF_3).

**Figure S1.** **(a)** Temperature of depended heat flow curves of samples with different cooling rates, under a heating rate of 40 K/min. **(b)** Temperature dependence of heat flow of as-quenched glasses and standard glasses of La_50_Ni_15_Co_2_Al_33_ MG at 40 K/min up-scan rate. The fictive temperature *T_f_* is determined by matching the shadow areas S_A_ and S_B_ through the energy-matching method for La_50_Ni_15_Co_2_Al_33_ MG with ribbon thickness of 40 μm [^2^](#_ENREF_2)^,^ [^3^](#_ENREF_3). **(c)** Dependence of fictive temperature on cooling rate for La_50_Ni_15_Co_2_Al_33_ MG.

**2. Additional data for the relation between shadow glass transition and β relaxation in glasses.**

For Au_49_Ag_5.5_Pd_2.3_Cu_26.9_Si_16.3_ MG, temperature dependence of the DMS at different frequencies for Au_49_Ag_5.5_Pd_2.3_Cu_26.9_Si_16.3_ MG from work of J.M. Pelletier *et al* [^4^](#_ENREF_4), and its β relaxation manifests as a weak but discernable peak merging into the tail of α relaxation. One can see that the FSC scan of Au_49_Ag_5.5_Pd_2.3_Cu_26.9_Si_16.3_ MG also exhibit a very pronounced endothermic peak (shadow glass transition) before *T_g_*. Interestingly, DSC scan of Au_49_Ag_5.5_Pd_2.3_Cu_26.9_Si_16.3_ MG exhibits also a less obvious but discernable endothermic peak at *T_g, shadow_* ~ 350 K, which is different with Pd_40_Cu_40_P_20_ MG. This may be due to its relatively lower *T_g_* than other MGs, which is caused by room temperature aging, and it’s inevitable, although we stored the sample in liquid nitrogen to prevent it from being aging at room temperature. Similarly, the corresponding relaxation map presented in Fig. S2 (d) for Au_49_Ag_5.5_Pd_2.3_Cu_26.9_Si_16.3_ MG, including shadow glass transition temperature *T_g, shadow_* from FSC curves at different heating rates and *T_β-relaxation_* from DMS at different testing frequencies. In addition, shadow glass transition temperature *T_g, shadow_* from conventional DSC curves at different heating rates is also included in Fig. S2 (d). For La_65_Ni_20_Al_15_ MG, a pronounced β relaxation peak was observed at *T_β-relaxation_* = 360 K for testing frequency of 8 Hz. Similarly, there is an obvious endothermic peak (shadow glass transition) at same temperature range on the FSC curve with heating rate of 40 K/s. However, replacing Ni by Cu atoms in La_65_Ni_20_Al_15_ MG makes β relaxation peak moves closer to the α relaxation, so the La_65_Cu_20_Al_15_ MG shows as a shoulder-like peak in the DMS. For La_65_Cu_20_Al_15_ MG, the normalized measured loss modulus *E’’*/*E’’_max_* is fitted with two Lorentzian functions to get *T_β-relaxation_* as showed in Fig. S4. For Ce_65_Ni_10_Al_25_ MG, only a broad hump peak is found in the DMS loss modulus curve. The Ce_65_Ni_18_Cu_2_Al_15_ MG and La_65_Cu_20_Al_15_ show similar β relaxation behavior with a slight change of peak temperature and intensity. Here, for these two Ce-based MGs, DMS curves only with testing frequency *f* = 1 Hz are displayed. In particular, for Pd_40_Ni_10_Cu_30_P_20_ MG, we tried to fit the normalized loss modulus *E’’*/*E’’_max_* with two functions, but neither the Gaussian function nor the Lorentz function succeeded. We speculate that this may be due to its complex relaxation processes at DMS [^5-9^](#_ENREF_5). Therefore, to get the peak temperature of β relaxation at different testing frequencies, we use the temperature of shallow glass transition (*T_g, shadow_*) to determine *T_β-relaxation_*, as showed in Fig. S6 (f).

**Figure S2. Experimental evidence for the relation between shadow glass transition and β relaxation in Au_49_Ag_5.5_Pd_2.3_Cu_26.9_Si_16.3_ MG.** **(a).** Comparison of heat flow curve at low heating rate (conventional DSC) and high heating rate (FSC). **(b).** Effect of heating rates on shadow glass transitions (endothermic peak) in Au_49_Ag_5.5_Pd_2.3_Cu_26.9_Si_16.3_ MG. Heat flow curves are measured by FSC using different heating rates from 3 K/s to 8000 K/s. **(c).** Temperature dependence of the DMS loss modulus (0.3 Hz) versus FSC heat flow (2 K/s) for Au_49_Ag_5.5_Pd_2.3_Cu_26.9_Si_16.3_ MG. **(d).** Relaxation map showing the β relaxation, α relaxation, shallow glass transition and real glass transition (*T_g_^overshoot^*) as a function of temperature. The hunter green line is a fitting using the Arrhenius equation.

**Figure S3. Experimental evidence for the relation between shallow glass transition and β relaxation in La_65_Ni_20_Al_15_ MG.** **(a).** The comparison of heat flow curves for a conventional DSC (0.33 K/s) and a FSC (500 K/s). **(b), (c).** Effect of heating rates on shadow glass transitions (endothermic peak). **(d).** The comparison of temperature dependence of the DMS normalized loss modulus (8 Hz) and FSC heat flow (40 K/s) for La_65_Ni_20_Al_15_ MG. **(e).** The temperature dependence of the normalized loss modulus *E’’*/*E’’_max_* for a La_65_Ni_20_Al_15_ MG, measured with frequency *f =* 0.5, 1, 2, 4, and 8 Hz, at the heating rate of 3 K/min. **(f).** Relaxation map showing the β relaxation, α relaxation, shadow glass transition and real glass transition (*T_g_*) as a function of temperature for La_65_Ni_20_Al_15_ MG. The green line is a fitting using the Arrhenius equation.

**Figure S4. Experimental evidence for the relation between shadow glass transition and β relaxation in La_65_Cu_20_Al_15_ MG. (a).** The comparison of heat flow curves for a conventional DSC (0.33 K/s) and a FSC (500 K/s). **(b), (c).** FSC heating scans as a function of the heating rate ranging from 20 to 8500 K/s for the La_65_Cu_20_Al_15_ MG. **(d).** The comparison of temperature dependence of the DMS loss modulus (4 Hz) and FSC heat flow (85 K/s) for La_65_Cu_20_Al_15_ MG. The normalized loss modulus *E’’*/*E’’_max_* (4 Hz) is fitted with two Lorentzian functions to get β relaxation peak temperature. **(e).** Temperature dependence of the normalized loss modulus *E’’*/*E’’_max_* for a La_65_Cu_20_Al_15_ MG, measured with frequency *f =* 0.5, 1, 2, 4, and 8 Hz, at the heating rate of 3 K/min. **(f).** Relaxation map showing the β relaxation, α relaxation, shadow glass transition and real glass transition (*T_g_*) as a function of temperature. The green line is a fitting using the Arrhenius equation.

**Figure S5. Experimental evidence for the relation between shadow glass transition and β relaxation in Ce_65_Ni_18_Cu_2_Al_15_ MG.** **(a).** Effect of heating rates on shadow glass transitions. **(b).** Temperature dependent normalized loss modulus *E’’*/*E’’_max_* of a Ce_65_Ni_18_Cu_2_Al_15_ MG. **(c).** The comparison of temperature dependence of the DMS loss modulus (8 Hz) and FSC heat flow (40 K/s) for La_65_Ni_18_Cu_2_Al_15_ MG. The normalized loss modulus *E’’*/*E’’_max_* (8 Hz) is fitted with two Lorentzian functions to get β relaxation peak temperature. **(d).** Relaxation map showing the β relaxation, α relaxation, shadow glass transition and real glass transition (*T_g_^overshoot^*) as a function of temperature. The green line is a fitting using the Arrhenius equation.

**Figure S6.** **Experimental evidence for the relation between shadow glass transition and β relaxation in Pd_40_Ni_10_Cu_30_P_20_ MG.** **(a).** The comparison of heat flow curves for a conventional DSC (0.33 K/s) and a FSC (2000 K/s). (b) and (c) Heat flow versus temperature at various heating rates from 20 to 10,000 K/s for the Pd_40_Ni_10_Cu_30_P_20_ MG. (d). Temperature dependence of the DMS loss modulus (4 Hz) versus FSC heat flow (300 K/s) for Pd_40_Ni_10_Cu_30_P_20_ MG. (e). Temperature dependent normalized loss modulus *E’’*/*E’’_max_* of a Pd_40_Ni_10_Cu_30_P_20_ MG. (f). Relaxation map showing the α relaxation, shadow glass transition and real glass transition (*T_g_*) as a function of temperature. The dashed lines are the heating rates at the corresponding frequencies.

**Figure S7.** Temperature dependence of the DMS loss modulus (1 Hz) versus FSC heat flow (500 K/s) for Ce_65_Ni_18_Cu_2_Al_15_ MG **(a)**, Zr_60_Ni_25_Al_15_ MG **(b)** and Zr_46_Cu_39_Al_8_Ag_7_ **(c)**.

**3.The shift factors (*n_s_*) for different composition metallic glasses.**

In order to match our DMS data with FSC data, we introduce a shift factor (*n_s_*) to convert different frequencies into different heating rates. According to the commonly used relation in experiment, the relaxation time is 100 s at the heating rate of *Q* = 20 K/min[^10^](#_ENREF_10), and the frequency is the inverse of relaxation time, so heating rates *Q* is proportional to frequency *f*, i.e., *Q* = *n_s_***f*. According to the equation: *Q* = *n_s_***f*, we shifted the α relaxation data points of the DMS to overlap the glass transition temperature at different heating rates by FSC measured, so we can get the shift factors (*n_s_*) of different materials by this method. The shift factors (*n_s_*) for the different materials are listed in table S1.

**Table S1.** **The shift factors (*n_s_*) for different composition materials.**

|  | Composition | *n_s_* |
| --- | --- | --- |
| 1 | Pd_40_Cu_40_P_20_ | 100 |
| 2 | Pd_40_Cu_30_Ni_10_P_20_ | 70 |
| 3 | La_50_Ni_15_Co_2_Al_33_ | 114 |
| 4 | La_65_Ni_20_Al_15_ | 5.6 |
| 5 | La_65_Cu_20_Al_15_ | 26.8 |
| 6 | Ce_65_Ni_18_Cu_2_Al_15_ | 4.6 |
| 7 | Au_49_Ag_5.5_Pd_2.3_Cu_26.9_Si_16.3_ | 6 |

**References for Supplementary Material**

1. Moynihan CT, Easteal AJ, De BOLT MA, Tucker J. Dependence of the fictive temperature of glass on cooling rate. *J. Am. Ceram. Soc.* 1976, **59**(1‐2)**:** 12-16.

2. Yue YZ, Christiansen Jd, Jensen SL. Determination of the fictive temperature for a hyperquenched glass. *Chem. Phys. Lett.*, **357**(1-2)**:** 20-24.

3. Liu YH, Fujita T, Aji DP, Matsuura M, Chen MW. Structural origins of Johari-Goldstein relaxation in a metallic glass. *Nat. Commun.* 2014, **5:** 3238.

4. Pelletier JM, Cardinal S, Qiao JC, Eisenbart M, Klotz UE. Main and secondary relaxations in an Au-based bulk metallic glass investigated by mechanical spectroscopy. *J. Alloy. Compd.* 2016, **684:** 530-536.

5. Yu HB, Wang WH, Bai HY, Samwer K. The β-relaxation in metallic glasses. *Natl. Sci. Rev.* 2014, **1**(3)**:** 429-461.

6. Wang Q, Zhang ST, Yang Y, Dong YD, Liu CT, Lu J. Unusual fast secondary relaxation in metallic glass. *Nat. Commun.* 2015, **6:** 7876.

7. Wang Q, Liu JJ, Ye YF, Liu TT, Wang S, Liu CT*, et al.* Universal secondary relaxation and unusual brittle-to-ductile transition in metallic glasses. *Mater. Today* 2017, **20**(6)**:** 293-300.

8. Sun Y, Peng SX, Yang Q, Zhang F, Yang MH, Wang CZ*, et al.* Predicting Complex Relaxation Processes in Metallic Glass. *Phys. Rev. Lett.* 2019, **123**(10)**:** 105701.

9. Wang Z, Sun BA, Bai HY, Wang WH. Evolution of hidden localized flow during glass-to-liquid transition in metallic glass. *Nat. Commun.* 2014, **5:** 5823.

10. Angell CA, Yue Y, Wang L-M, Copley JRD, Borick S, Mossa S. Potential energy, relaxation, vibrational dynamics and the boson peak, of hyperquenched glasses. *J. Phys.: Condens. Matter*, **15**(11)**:** S1051-S1068.
